# Fatigue-Assisted Grain Growth in Al Alloys

**DOI:** 10.1038/s41598-017-10889-8

**Published:** 2017-08-31

**Authors:** R. Goswami, C. R. Feng, S. B. Qadri, C. S. Pande

**Affiliations:** Multifunctional Materials, Materials Science and Technology Division, Naval Research Laboratory, Washington DC, 20375 USA

## Abstract

Stress-assisted grain growth at room temperature is known for materials with nanocrystalline grains. For larger grain sizes, the grain growth usually takes place at higher homologous temperatures even under stress. Here we report, for the first time, significant grain growth at room temperature under fatigue loading in microcrystalline grains (≥10 μm) in Al 7075. We demonstrate that this grain growth at room temperature is similar to non-uniform grain growth due to grain rotation and coalescence rather than the thermally and the stress-assisted driven grain growth. We show that the grain growth is associated with the formation of a strong near-Cu {112}<111> texture component as a result of fatigue-assisted deformation. These changes in microstructural features (viz., grain size, grain orientations and texture) are fundamentally important in understanding the cyclic crack induced deformation behavior and for predicting the fatigue lifetime in structural materials.

## Introduction

Grain growth in polycrystalline materials can be curvature driven^1–3^, or by coalescence of subgrains^4, 5^. Recently, for nanocrystalline grains^6, 7^, it has been reported that the grain growth is mediated by grain rotation. The grain growth can also be driven by stress at low homologous temperatures. Considerable grain growth has been observed in nanocrystalline metals at room temperature under indentation^8, 9^, compression^10^, tensile^11–14^ and fatigue loadings^15–19^. As the shear stress drives the dislocation motion, grain boundaries, particularly, low-angle grain boundaries, consisting of dislocation arrays, can migrate under the shear stress due to the collective motion of dislocations. Cahn and Taylor^20^ proposed the theory of coupled high-angle grain boundary motion under stress. They predicted that the migration of a grain boundary can generate a coupled tangential motion of the two grains relative to each other, and this tangential motion is proportional to the normal motion of the boundary, and as a result of such translations, grain rotations can occur. Thus, in relatively large grains, the migration of grain boundaries can also be stress driven. Recently, Winning *et al*.^21^ conducted the stress-assisted growth experiments on micron sized Al bi-crystal with different tilt boundaries, and showed that the motion of high-angle grain boundaries (<112> and <111> tilt boundaries) under stress is a thermally activated process. However, the grain growth close to room temperature in microcrystalline grains under stress, possibly due to a crack stress field, has not been observed before.

Here we demonstrate, for the first time, significant grain coarsening at room temperature on polycrystalline micron sized grains in Al alloys under fatigue loading. Such grain coarsening has a major effect on fatigue life prediction. We show using high- resolution electron back scattered diffraction (EBSD) and X-ray diffraction (XRD) that the grain coarsening process is the result of lattice rotation and coalescence during fatigue rather than the thermally driven grain boundary migration.

## Results and Discussion

### Grain growth under fatigue loading

**Figure 1:**
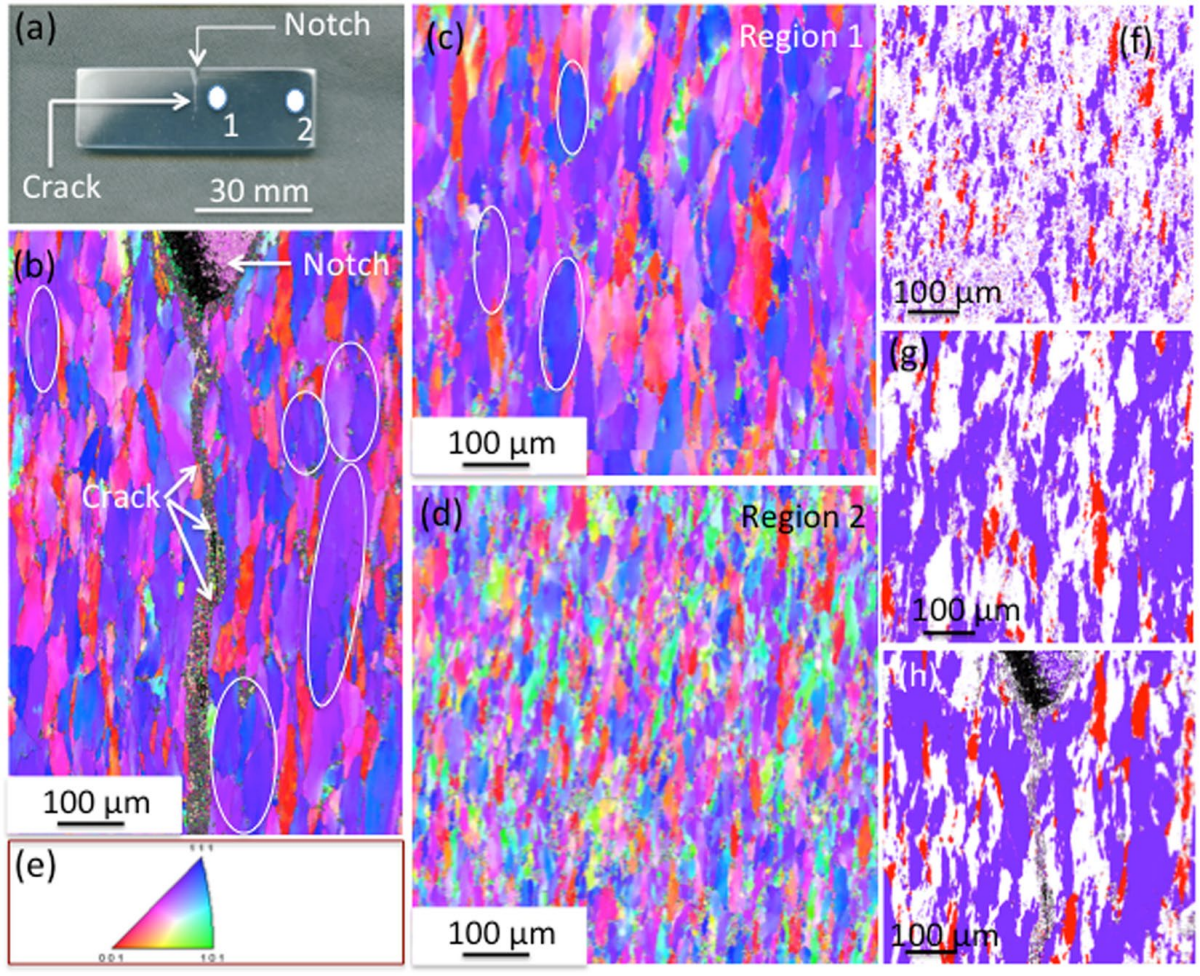
EBSD maps showing grain growth. (**a**) A part of the compact tension (CT) specimen showing the crack from the notch corresponding to ΔK = 13 MPa m^1/2^. (**b**) An inverse pole figure image showing the pancake sized grains at either side of the crack. (**c**) An inverse pole figure image showing the pancake sized grains at 5 mm from the crack. The grain size and morphology look similar to the size and morphology close to the crack. (**d**) An inverse pole figure image showing the pancake sized grains at 25 mm from the crack. A significant change in grain size could be observed. (**e**) The inverse pole figure showing the color coding. **(f-h)** The crystal direction maps (obtained with the <112> and <001> orientation at 10° tolerance), close to the crack, 5 mm and 25 mm away from the crack, respectively, showing grain coalescence in number of regions within 5 mm from crack, compered to the 25 mm away from the crack.

Significant grain coarsening was observed around the fatigue crack, within 10–12 mm from the crack (see Fig. 1). The part of the fatigue specimen containing the fatigue crack is shown in Fig. 1(a). The grain size and grain orientation around the crack were obtained using the inverse pole figure (IPF) at several regions, close to and away from the crack. Figure 1(b–c) are the inverse pole figure (IPF) maps showing larger sized grains close to the crack (0 to 200 μm at either side of the crack), and 5 mm away (region 1 in Fig. 1(a)) from the crack, respectively, as compared to the grain size at 25 mm away (region 2) from the crack (see Fig. 1(d)). Some grains were observed to be significantly wider (50 to 80 μm) and longer (100–200 μm), as indicated by ellipses, compared to the average width and length of 12 and 80 μm, respectively, around 25 mm away from the crack. IPF maps show these wider grains contain low-angle grain boundaries. To delineate the grain growth clearly, we generated (see Fig. 1(f–h)) the crystal direction maps close to the <112> (purple regions) and <001> orientations (red regions) at 10° tolerance. Figure 1(g,h) show about 50% <112> oriented grains in regions within 0 to 5 mm from the crack, with a 10° tolerance, as compared to about 25% <112> oriented grains with similar tolerance 25 mm away from the crack (see Fig. 1(f)).

**Figure 2:**
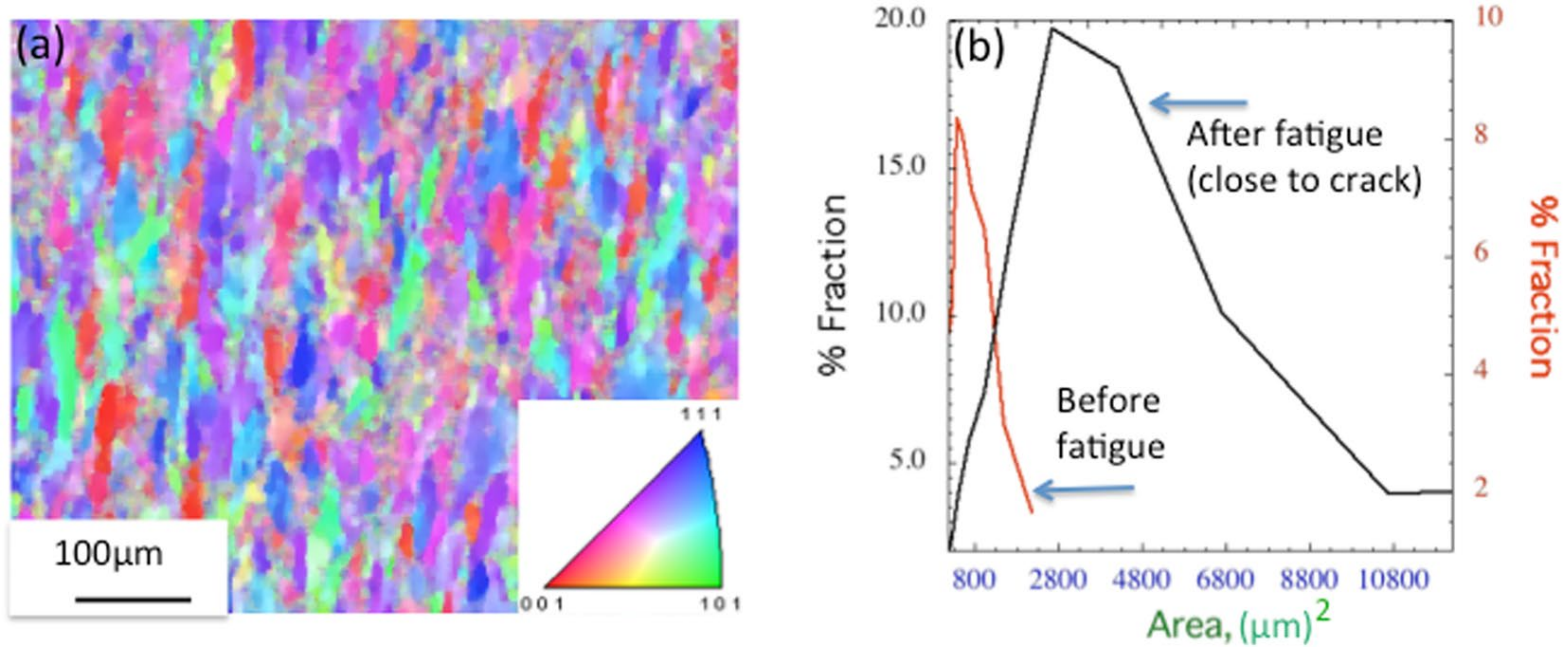
IPF showing the size and orientation of the initial specimen before fatigue. (**a**) The IPF of the initial specimen in T7 condition before fatigue. (**b**) The size distribution before and after fatigue, showing the grain growth.

The initial microstructure shows that grains are elongated at the T7 condition (Fig. 2(a)). The crystal direction map obtained from the inverse pole figure, as shown in Fig. 2(a), shows that the initial specimen before fatigue contains about 25.8% of <112> oriented grains (see the Supplementary Figure). Details of grain orientations of the initial specimen before fatigue, showing the pole figures and the texture information, have been given as Supplementary Figure. The size distribution (see Fig. 2(b)) of the initial specimen before fatigue loading and the specimen after fatigue loading (close to the crack) is given for comparison. After fatigue, the distribution becomes more skewed to the right, and the mean increase in area is approximately 366%, suggesting a significant increase in size after the fatigue loading. As the grains are elongated, the size is presented here in terms of area.

The grain growth observed in our case under fatigue conditions has occurred in somewhat non-uniform fashion. It cannot obviously be characterized as the normal grain growth, where the average grain size grows in a slow uniform manner under the influence of thermal conductions and grain boundary curvature, as well as an abnormal grain growth, where a subset of favored grains grow rapidly at the expense of their neighbors, ultimately resulting in a bimodal distribution. Such a bimodal distribution has not been observed in the present investigation (see Fig. 2(b) for size distribution). Although the gain size has increased, the grain morphology (pancake-like) remains similar, suggesting that the grains have not been recrystallized as a result of crack growth. Note that the applied stress is perpendicular to the long axis direction of the grain, and the crack is running parallel to the long axis direction. This is manifested in maintaining the elongated direction of the grain. Note also that in the recrystallized state, an elongated grain will be replaced by relatively stress free equiaxed small polycrystalline grains, which contain considerably lower dislocation density as compared to the T7 tempered state. However, the elongated grains close to the crack have a higher dislocation density, as estimated from the line broadening of the 111 peak by XRD compared to the grains away from the crack.

### Texture formation and lattice rotation around the crack

**Figure 3:**
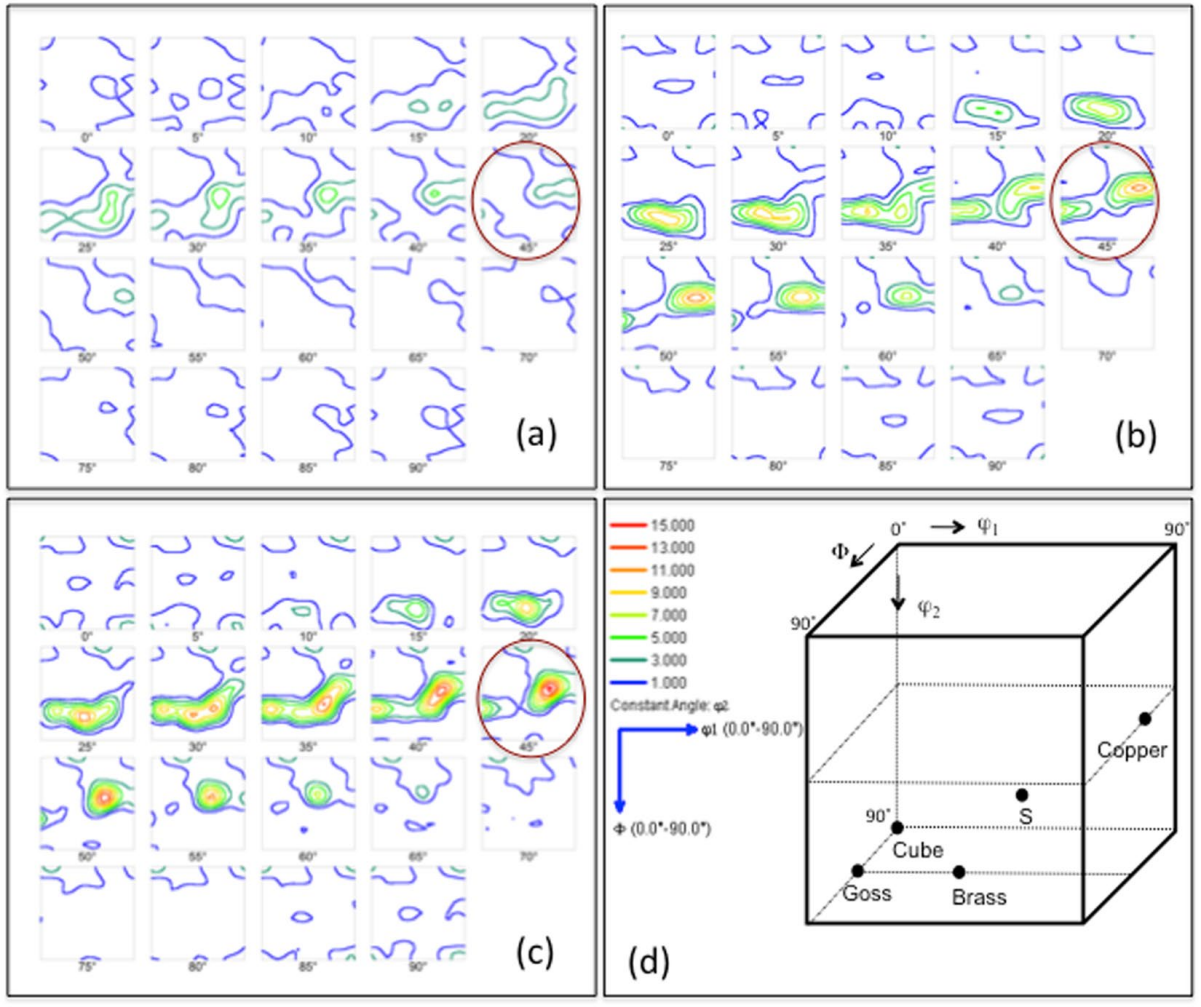
The ODF sections parallel to φ2 from 0–90° with an increment of 5° showing texture formation and grain rotation. (**a**) ODF sections 25 mm away from crack and (**b**) ODF sections 5 mm away from crack. (**c**) ODF sections close to the crack. A strong near {112} <111> Cu texture component, corresponding to φ1, φ, φ2 of 80°, 35° and 45°, respectively, develops within 5 mm from crack. The φ2 = 45° sections for all positions have been indicated by circles. (**d**) A schematic diagram showing the Euler coordinates, φ1, φ, φ2, for ideal cube, goss, brass, S and copper texture in FCC materials. Inset shows the intensity level, and φ1 and φ directions for Fig. 3(a–c).

In addition to the grain growth, we observe a significant development of a near Cu-type texture component, {112} <111> Cu, within 0 to 5 mm from the crack. Texture is usually represented by the orientation distribution function (ODF) and three Euler angles, φ1, φ and φ2. The ODF maps have been generated by sectioning through the Euler space from the IPF. The ODF sections parallel to φ2 from 0–90° with 5° increment are shown in Fig. 3(a–c). The ODF sections obtained from the inverse pole figure 25 mm away from the crack (see Fig. 3(a)) show no prominent texture component. As we move closer to the crack, within 5 mm, a strong near {112} <111> Cu texture component, corresponding to φ1, φ, φ2 of 80°, 35° and 45°, respectively. The φ2 = 45° sections for all positions have been indicated by circles. The Cu-type texture usually develops during deformation of FCC materials^22^, particularly, relatively high stacking fault energy materials, such as Al, Ni or Cu, develop this type of texture during deformation. However, the recrystallization texture of cold rolled Al alloys sheet is predominantly cube ({100} <001>). The position of all texture components, such as {001} <100> cube and {011} <100> goss, {112} <111> copper, {123} <634> S, and {011} <211> brass in Euler space is shown in Fig. 3(d). The ODF sections of the initial specimen before fatigue (see the Supplementary Figure) obtained from the inverse pole figure, as shown in Fig. 2(a), show no prominent texture component.

**Figure 4:**
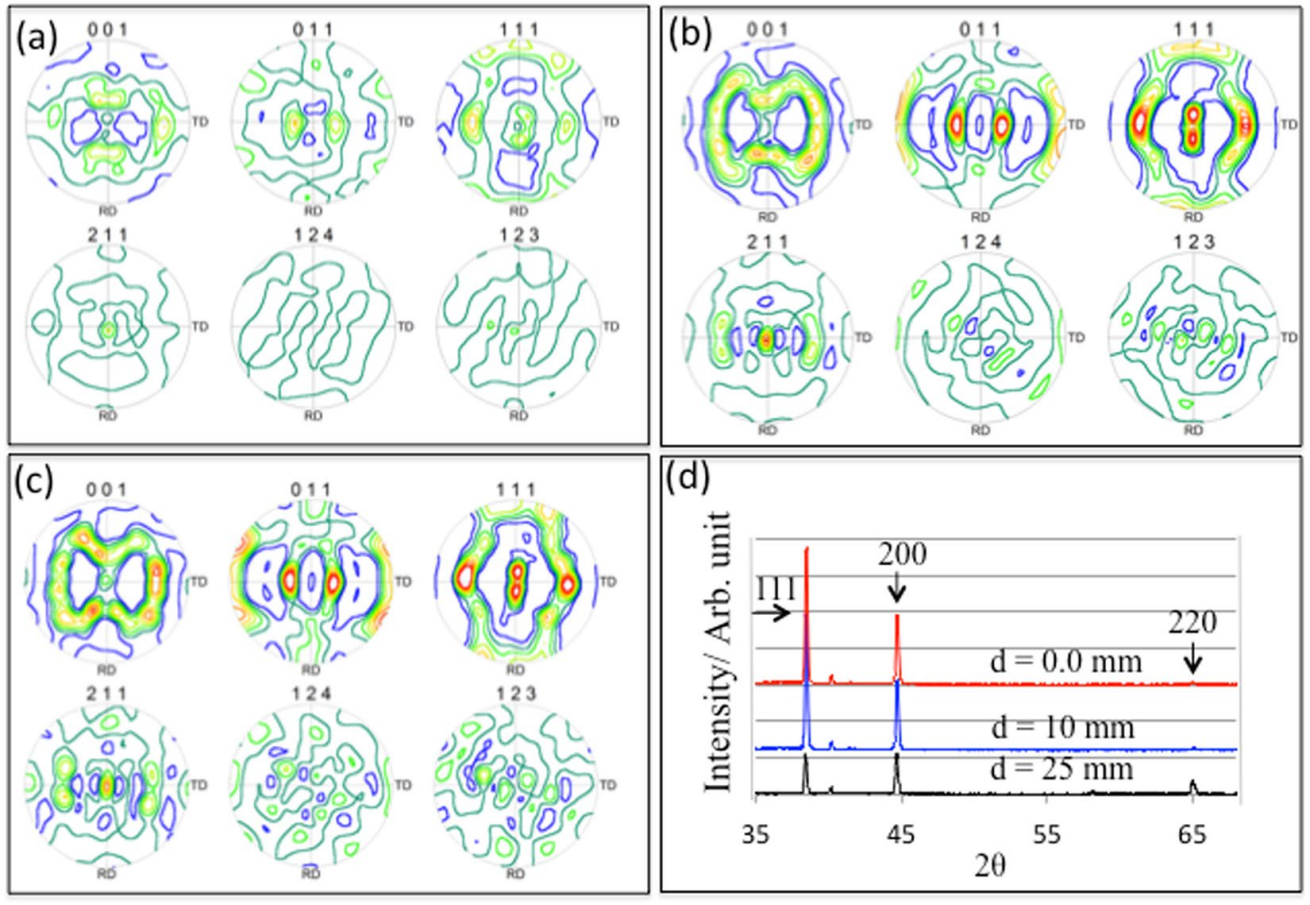
The pole figures showing grain orientation close to [001] and [112] poles. (**a**) The poles close to the crack (**b**) the poles 5 mm away from crack and (**c**) the poles 25 mm away from crack. (**d**) A series of XRD diffraction patterns at different locations, close to the crack, 5 mm and 25 mm away from the crack, showing the relative variation of 111 002 and 220 peaks.

The grain orientations can also be represented by pole figures (PFs). The PFs of <001>, <011>, <111>, <211>, <124> and <123> close to the crack, 5 mm and 25 mm away from the crack are shown in Fig. 4(a–c), suggesting that the <111> pole and <112> are much stronger as we move closer, within 5 mm, to the crack. Figure 4(d) shows a series of x-ray diffraction (XRD) patterns obtained from similar locations of the sample (see Fig. 1(a)), close to the crack and 5 and 25 mm away from the crack, showing the relative variations of 111 and 200 Al peaks. The intensity of 111 and 200 peaks are approximately equal 25 mm away from the crack, suggesting that more grains are 200 oriented. The peak ratio of 111 to 200 intensity increases from 0.8 at 25 mm from the crack to 2.2 close to the crack, implying that the change of peak ratio is approximately 175%, which is consistent with the pole figures.

From these results, presented above, it can be concluded that the strong near {112} <111> Cu texture develops as a result of plastic deformation around the crack. It is well known that in metals and alloys, plastic deformation leads to texture formation, which involves grain rotations. The formation of strong near-Cu type texture, thus, indicates considerable lattice rotation across several grains around the crack as a result of fatigue crack growth at room temperature. This is consistent with the recently reported observations on fatigue-mediated lattice rotation in Al alloys^23^.

### Softening behavior

**Figure 5:**
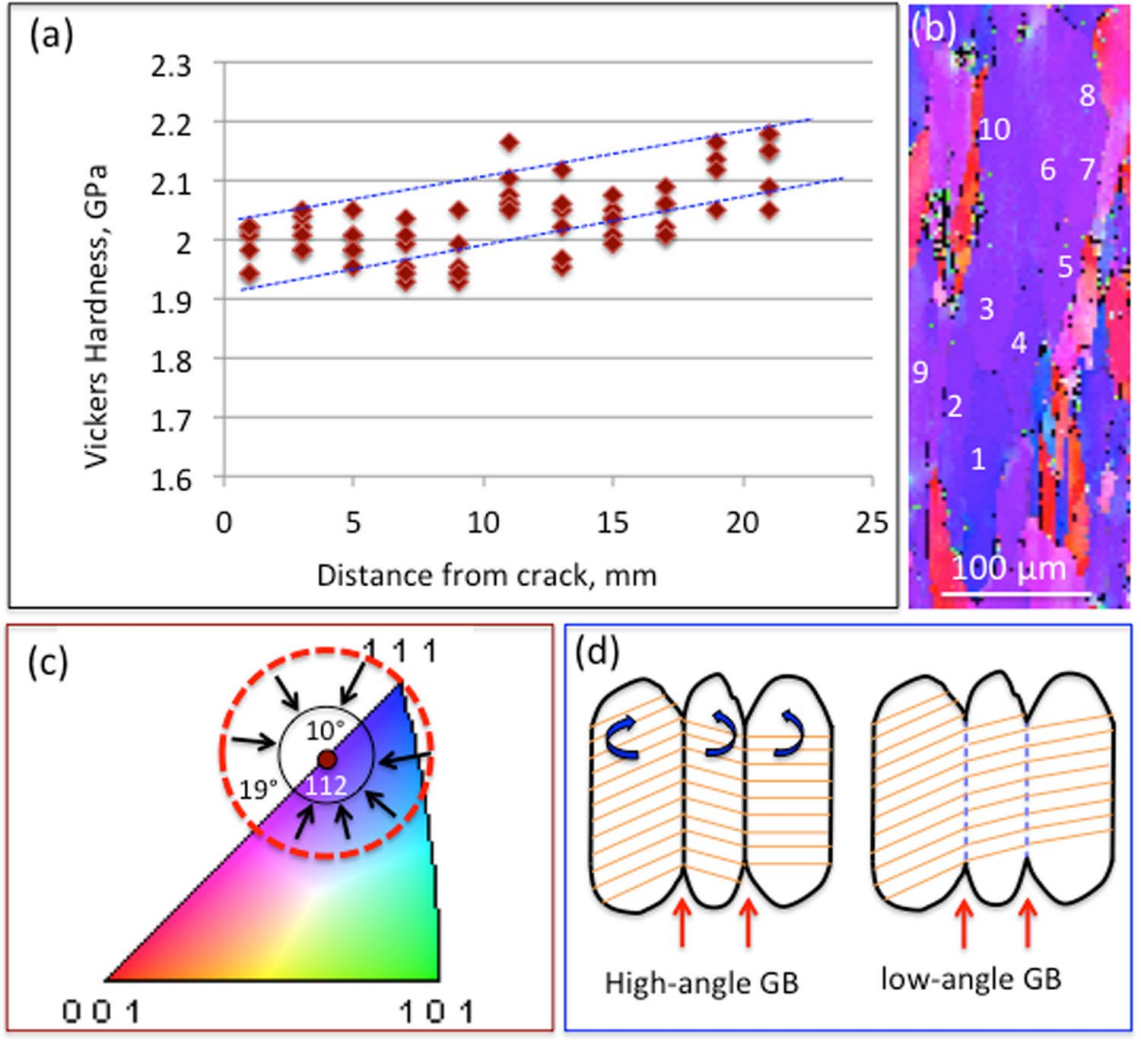
Diagrams showing mechanical behavior, and mechanism of grain coalescence and grain growth. (**a**) Vickers hardness as a function of distance from the crack showing the hardness decreases as we approach the crack. A band with dotted lines is drawn to show the increase in hardness with distance. (**b**) An IPF image, showing the coalescence of at least 10 grains close to a <112> orientation. (**c**) A color triangle showings grain orientations that are 10–19° apart from <112> orientation can be rotated into <112> with 10° tolerance by 9° rotation. (**d**) Schematic diagrams showing that three grains with high-angle grain boundaries become a single grain containing low-angle grain boundaries through lattice rotation and coalescence.

We observe that regions close to the crack are softer (see Fig. 5(a)). The observed decrease in hardness close to the crack is around 10%, which is due to grain coarsening. One would expect considerable drop in the hardness/strength (≈ 41%) close to the crack according to the Hall-Petch softening, as the average increase in grain size (see Fig. 1) is approximately twice. However, the loss of hardness observed due to coarsening is 10%, suggesting that the other microstructural features, increase in dislocation density and low angle boundaries, contribute to increase in the hardness/strength to a significant extent. In fact, the average increase in dislocation density has been estimated as 30% (within ~12 mm from the crack) using the line broadening^23, 24^ of the 111 peak.

### Mechanism of grain growth

In cyclic loading grains rotate toward the loading axis^23^. The magnitude of lattice rotation can be calculated by knowing the amount of dislocation gliding through the slip planes. Assuming that the total number of dislocations produced during crack growth contributes to the total amount of displacement due to slip; an average rotation angle, θ, can be given as:

1 $${\rm{\theta }}={\tan }^{-1}({\text{nb}/{\rm{l}}}_{{\rm{w}}})$$

where n is the total number of dislocations, l_w_ is the average width of the grain and b is the Burgers vector. With an increase of dislocation density of 30%, the corresponding increase of dislocation density is 6.5 × 10^12^ m^−2^. This represents roughly 0.6 × 10^4^ dislocations for a 80μm × 12μm pancake size grain. For the initial average grain width, l_w_, of 12 μm, the estimated average angle of rotation is ≈ 9°. Another factor that could contribute to grain rotation is due to higher grain boundary (GB) dislocation content^25^. The increase in grain boundary dislocation content alters the GB angle between neighboring grains due to the well-known Frank–Bilby equation^25^.

The results on EBSD studies, IPF, ODM, PF, and the X-ray diffraction observations presented here allow us to define a broad outline of a mechanism. Fatigue crack growth in the in the material results in cycle-dependent plastic deformation due to dislocation motion, which gives higher magnitude of dislocation density and also indicates that dislocation motion is important. It is likely that the cyclic/reverse slip of dislocations near the crack tip can cause higher stresses and dislocation damage accumulation. This together with slip constraints, leads to grain rotation and coalescence; thereby increasing the grain size. Figure 5(b) is the IPF image, obtained from Fig. 1(b), showing the coalescence of at least 10 grains close to a <112> orientation. The internal boundaries, which become low angle boundaries, of the coalesced grains can still be observed. Note, the estimated average lattice/grain rotation from the increase in dislocation density is 9°, which is sufficient to bring number of grains within 10° tolerance of <112> (see Fig. 5(c)). The grain rotation and the subsequent coalescence between three grains have been shown schematically in Fig. 5(d). This rotation, driven by a thermodynamic reduction of the total grain boundary energy of the system, will eventually diminish as the energy is lowered leading to grains properly orientated for coalescence. This would explain the experimental observation of grain rotation and grain growth. Thus, in the present case, grain coarsening observed by us is not a thermally driven and stress-assisted grain growth mechanism but better described by a non-uniform grain growth due to dislocation accumulation; resulting in grain rotation and growth.

## Summary and Conclusions

In summary, we have observed significant grain growth in the plastic zone mediated by fatigue crack growth at room temperature in Al 7075. This grain growth is neither thermally driven nor thermal stress-assisted driven, but more akin to non-uniform grain growth due to dislocation accumulation resulting in grain rotation and grain growth. Such grain rotation in the plastic zone leads to the formation of the near-Cu texture, {112} <111>. This result provides a new insight of the underlying physics of the deformation process under the fatigue loading.

## Method

Fatigue crack growth tests were performed along short transverse (ST) direction in vacuum (<6 × 10^–6^ Pa) background pressure at a cyclic load frequency of 10 Hz using a sine waveform and a load ratio of 0.10 on compact tension (CT) specimen, 80 mm in length, 60 mm in width and 10 mm in thickness, made of cold rolled-Al 7075-(T7) alloy. The T7 tempered condition corresponds to the treatment, solution heat treated then overaged/stabilized. The part of the CT specimen containing a fatigue crack is shown in Fig. 1(a). Grains were observed to be elongated or pancake shaped parallel to the rolling direction. In order to study the characteristics of plastic zone and the dislocation configurations, the crack growth was arrested at a different crack lengths corresponding to a certain difference in stress intensity factor (ΔK) close to the Paris regime (ΔK = 13 MPa m^1/2^). X-ray diffractions were then obtained using a Rigaku 18 kW generator and a high resolution powder diffractometer perpendicular to the crack length at number of locations in the CT specimen. The locations are close to the crack, and 5, 10, 15, 20 and 25 mm from the crack on both sides of the crack. The spot size of 1 mm wide window was used to collect the data at different locations. For EBSD, a JEOL JSM-7001F SEM operating at 30 kV and equipped with a TSL/EDX Hikari EBSD detector was used to acquire the data with a step size of 1.0 µm. Analysis of the EBSD data and grain size was performed with the TSL/EDX OIM Analysis-5.31 software package. To investigate the mechanical behavior, micro-indentation hardness tests were performed to obtain the hardness values as a function of distance from the crack. We use Vickers tip at 100 g of load and dwell time of 15 seconds, and obtain 5 indents at each location for each sample.

## Electronic supplementary material

Supplementary Information
