# Supplementary material for: Fatigue-Assisted Grain Growth in Al Alloys
Source: Sci Rep. 2017 Aug 31;7:10179. doi: 10.1038/s41598-017-10889-8 (PMC5579296; doi:10.1038/s41598-017-10889-8)
Supplement: Supplementary file 1 — Supplementary Information [file 41598_2017_10889_MOESM1_ESM.pdf]

Fatigue-Assisted Grain Growth in Al Alloys

R. Goswami, C.R. Feng, S. B. Qadri and C.S. Pande

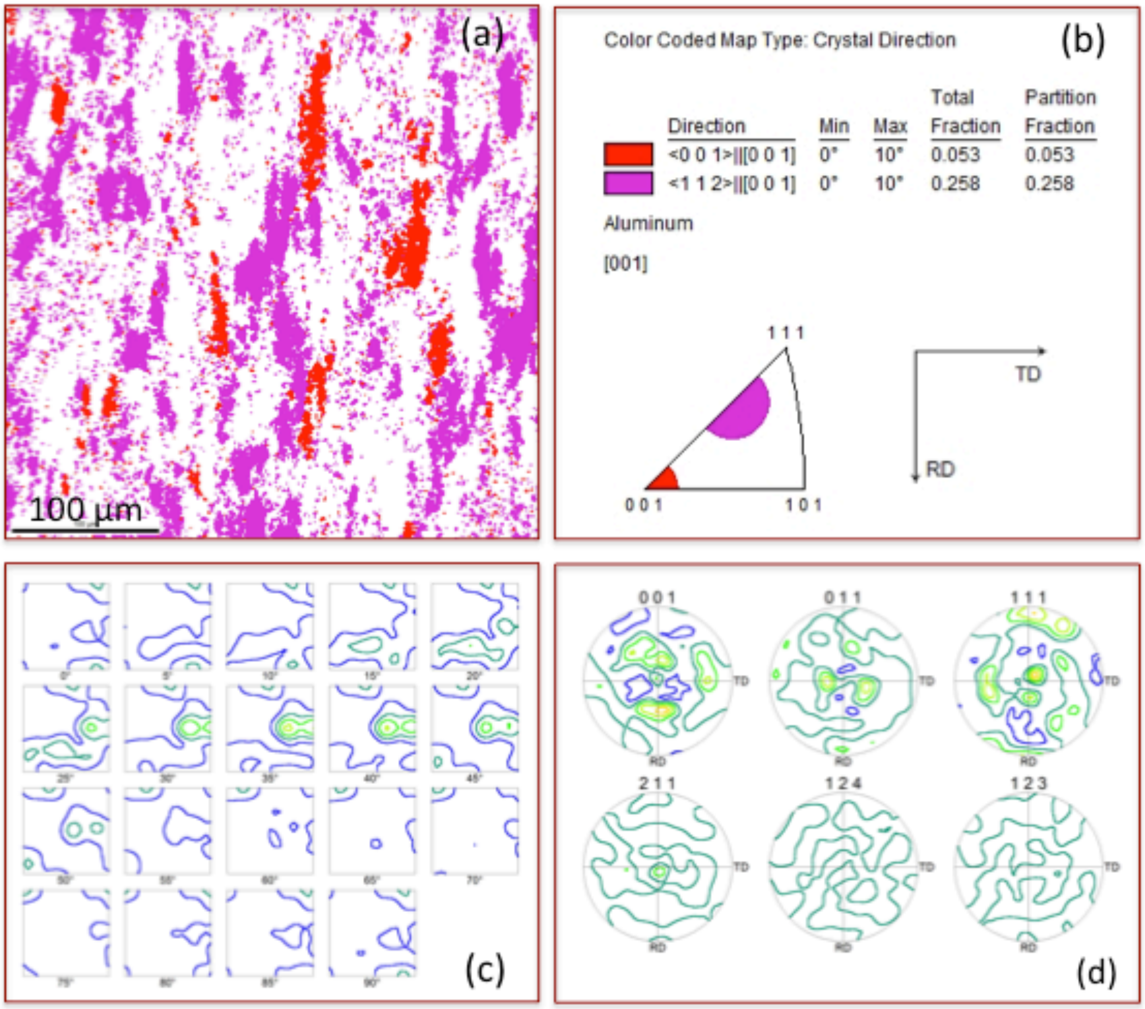

Orientation and texture information of specimen before fatigue loading:

a) The crystal direction map (obtained with the <112> and <001> orientation at 10° tolerance of the initial specimen before fatigue. b) The orientation information showing about 25.8% grains are <112> oriented. c) The ODF sections of the initial specimen before fatigue parallel to φ2 from 0-90° with an increment of 5° showing no prominent texture component. d) The grain orientations before fatigue are shown by pole figures of <001>, <011>, <111>, <211>, <124> and <123>.
